# PSA testing and receipt of curative treatment in elderly Japanese men with prostate cancer: a population-based study

**DOI:** 10.1186/s12894-026-02266-3

**Published:** 2026-07-27

**Authors:** Shohei Tobu, Hirokazu Takahashi, Hiroshi Isoda, Kenji Fujimoto, Mitsuru Noguchi

**Affiliations:** 1 Department of Urology, Faculty of Medicine, Saga University, Nabeshima, 5-1-1 Nabeshima, Saga City, Saga 849-8501 Japan; 2 Division of Metabolism and Endocrinology, Faculty of Medicine, Saga University, 5-1-1 Nabeshima, Saga City, Saga 849-8501 Japan; 3 Liver Center, Saga University Hospital, 5-1-1 Nabeshima, Saga City, Saga 849-8501 Japan; 4 Occupational Health Data Science Centre, Department of Preventive Medicine and Community Health, University of Occupational and Environmental Health, 1-1, Iseigaoka, Yahatanishiku, Kitakyushu, 807-8555 Japan

**Keywords:** Prostate cancer, Prostate-specific antigen, PSA testing, Elderly, Curative treatment, Population-based study

## Abstract

**Objective:**

This study aimed to evaluate whether regular prostate-specific antigen (PSA) testing is associated with the receipt of curative treatment for prostate cancer among men in Japan and to identify associated factors.

**Methods:**

A retrospective analysis was conducted using the claims data from the National Health Insurance and Late-Stage Elderly Health Care System in Saga Prefecture between March 2014 and March 2022. Patients were categorized into PSA testing (≥ 1 test ≥ 6 months before treatment) and non-PSA testing groups. Curative treatment was defined as either a radical prostatectomy or carbon-ion radiotherapy. Multivariable logistic regression was used to identify the factors associated with curative treatment.

**Results:**

Among 2,200 patients, 737 (33.5%) underwent PSA testing, and 708 (32.2%) received curative treatment. Patients who underwent PSA testing were older and more likely to participate in Specific Health Checkups. The proportion receiving curative treatment was higher in the PSA testing group (36.4% vs. 30.1%, *p* < 0.001). Age (OR = 0.85), PSA testing (OR = 1.53), and participation in Specific Health Checkups, which do not include PSA testing, were independently associated with curative treatment (OR = 1.64).

**Conclusions:**

PSA testing was associated with a higher likelihood of receiving curative treatment for prostate cancer. Participation in Specific Health Checkups may reflect a population more engaged in preventive healthcare or with greater access to screening opportunities.

**Supplementary Information:**

The online version contains supplementary material available at https://doi.org/10.1186/s12894-026-02266-3.

## Introduction

Prostate cancer is a common malignancy among elderly men in Japan, with both the incidence and mortality rates showing increasing trends [1–3]. Therefore, early diagnosis and appropriate treatment planning are crucial for improving patient outcomes. Prostate-specific antigen (PSA) testing is widely used as a noninvasive and convenient screening method [4–6]. Several international guidelines, including those of the European Association of Urology (EAU), recommend risk-adapted PSA testing after shared decision-making, whereas the U.S. Preventive Services Task Force (USPSTF) recommends individualized decision-making for men aged 55–69 years and discourages routine screening in older men. However, in Japan, PSA testing is conducted only as part of optional health screenings or limited municipal health checkups, and a nationwide population-based PSA screening program has not been implemented. As a result, PSA testing rates vary substantially depending on individual health awareness and access to screening opportunities.

PSA testing in elderly men is expected to increase opportunities for early diagnosis of prostate cancer and, consequently, increase the proportion of patients eligible for curative treatments, such as radical prostatectomy or radiotherapy. Earlier detection through PSA testing may increase the likelihood of diagnosing localized disease before progression to metastatic disease, thereby expanding the opportunity to receive treatment with curative intent. However, given the typically slow progression of prostate cancer, many elderly patients may die from other causes without requiring treatment [7, 8]. From this perspective, the cost-effectiveness of PSA-based screening remains controversial [9]. Despite ongoing debate, empirical evidence evaluating the clinical impact of routine PSA testing in elderly men in Japan remains limited, and definitive conclusions have yet to be reached.

In this study, we retrospectively analyzed the medical and screening claims data from the National Health Insurance and Late-Stage Elderly Health Care System in Saga Prefecture. We aimed to evaluate whether regular prediagnosis PSA testing behavior is associated with the likelihood of receiving curative treatment for prostate cancer.

## Material and methods

### Data sources

This retrospective observational study was conducted using medical and participation in Specific Health Checkups claims data from men enrolled in the National Health Insurance or the Late-Stage Elderly Health Care System in Saga Prefecture, Japan, between March 2014 and March 2022.

### Definition of diagnosis, examination and treatment

In the claims data, the disease diagnosis was defined by referring to disease codes, and examinations and treatments were identified using medical intervention codes (Supplementary Table 1). In this study, prostate cancer was defined as the presence of a diagnosis of prostate cancer, a record of undergoing prostate biopsy, and having received treatment for it. Curative treatments for prostate cancer included surgery and carbon ion radiotherapy, whereas non-curative treatments included hormone therapy and chemotherapy. In cases where both curative and non-curative treatments were administered, if hormone therapy or chemotherapy preceded curative treatment, it was considered neoadjuvant therapy and thus was included as part of the curative treatment. However, if non-curative treatment was administered after curative treatment, these cases were excluded from the study because of the inability to distinguish between adjuvant therapy and recurrence or residual disease. Other covariates, including age and comorbidities such as hypertension, diabetes mellitus, and dyslipidemia, were defined using ICD-10 codes (hypertension: I10, I11, I13; diabetes mellitus: E11–E14; dyslipidemia: E79.0). Data on participation in Specific Health Checkups were also collected. These checkups are insurer-based screenings for lifestyle-related diseases mandated by Japanese law, which do not include PSA testing. Patients managed with active surveillance or watchful waiting without treatment claims were not included in the study cohort.

### Definition of PSA testing

The index date was defined as the date of initiation of prostate cancer treatment. PSA measurements taken after the initiation of treatment are unlikely to reflect prostate cancer surveillance. To minimize inclusion of PSA testing performed during the diagnostic work-up immediately preceding treatment, a 6-month washout period was applied. Therefore, in this study, we evaluated the presence and frequency of PSA testing within the period from the earliest available claims record to six months prior to the initiation of prostate cancer treatment. If at least one PSA test was identified in the claims data during this period, the patient was classified as having undergone pre-treatment PSA testing. The frequency of PSA testing was calculated by dividing the number of PSA tests by the duration of the observation period (years). In contrast, the absence of PSA testing claims during this period was defined as non-PSA testing. PSA tests performed within six months prior to prostate cancer treatment and those performed after prostate cancer treatment were not evaluated in this analysis. There were no missing data for the variables included in the primary analyses.

### Statistics

Chi-square tests and multivariate logistic regression analyses were performed to evaluate the association between PSA testing and the receipt of curative treatments. Multiple comparisons were adjusted using Bonferroni correction. In the logistic regression analysis, PSA testing was used as an explanatory variable in Model 1, and PSA testing frequency was used in Model 2. Covariates included all clinically relevant variables available in the claims database, namely age at diagnosis, hypertension, diabetes mellitus, dyslipidemia, history of participation in the Specific Health Checkup program, and PSA testing (Model 1) or PSA testing frequency (Model 2). All statistical analyses were conducted using JMP Pro 14 (SAS Institute Inc., Cary, NC, USA), and a *p*-value < 0.05 was considered statistically significant. This study was approved by the Ethics Committee of Saga University Faculty of Medicine (Approval No. 2024-08-R-02). The requirement for informed consent was waived due to the retrospective nature of the study and the use of anonymized data.

## Results

### Patient characteristics

**Fig. 1:**
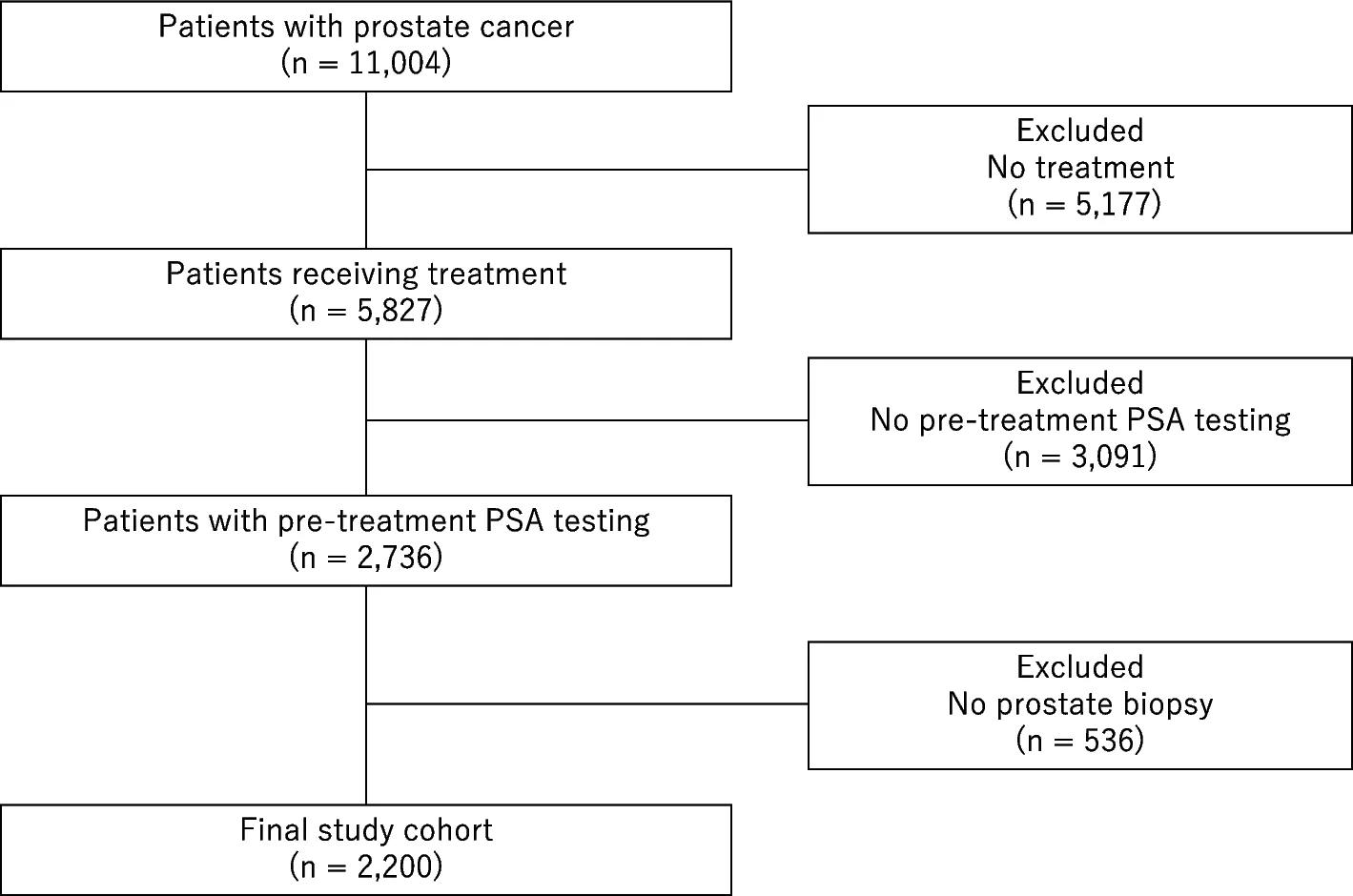
Flow diagram of patient selection. Among 11,004 patients with prostate cancer identified in the claims database, patients without treatment, without pre-treatment PSA testing, or without prostate biopsy were sequentially excluded, resulting in a final study cohort of 2,200 patients

The patient selection process is shown in Fig. 1. Among 11,004 patients with prostate cancer identified in the claims database, patients without treatment, without pre-treatment PSA testing, or without prostate biopsy were sequentially excluded, resulting in a final study cohort of 2,200 patients. The baseline characteristics of the study population are summarized in Table 1. The mean age at diagnosis was 74.6 years, and the prevalence of common comorbidities, including hypertension, diabetes, and dyslipidemia, was 75.7%, 48.8%, and 55.0%, respectively. A total of 737 patients (33.5%) underwent PSA testing, and 728 (33.1%) had a history of participation in the Specific Health Checkup program. Overall, 708 patients (32.2%) received curative treatment, whereas 1,492 (67.8%) received non-curative treatment.

**Table 1:** Characteristics of the patients. This table summarizes the baseline characteristics of 2,200 elderly male patients with prostate cancer who had a history of treatment identified from claims data. The variables included the age at diagnosis, the presence of comorbidities (hypertension, diabetes, dyslipidemia), history of specific health checkups, PSA testing status, and frequency of PSA testing. Treatment modalities were classified as curative or non-curative. Non-curative treatment was further categorized into systemic therapy only and adjuvant or recurrent treatment

Variables	
Male/female, *n*	2200/0
Age at diagnosis of prostate cancer, years	74.6 ± 6.6
Hypertension, *n* (%)	1665 (75.7)
Diabetes, *n* (%)	1074 (48.8)
Dyslipidemia, *n* (%)	1211 (55)
History of Specific Health Checkup, *n* (%)	728 (33.1)
PSA testing, *n* (%)	737 (33.5)
PSA testing frequencies, n/year	0.9 ± 1.6
Curative treatment, *n* (%)	708 (32.2)
Non-curative treatment, *n* (%)	1492(67.8)

Continuous data is shown as mean ± standard deviation

*Abbreviation*: *PSA* prostate-specific antigen

### Characteristics of the patients with or without PSA testing

**Fig. 2:**
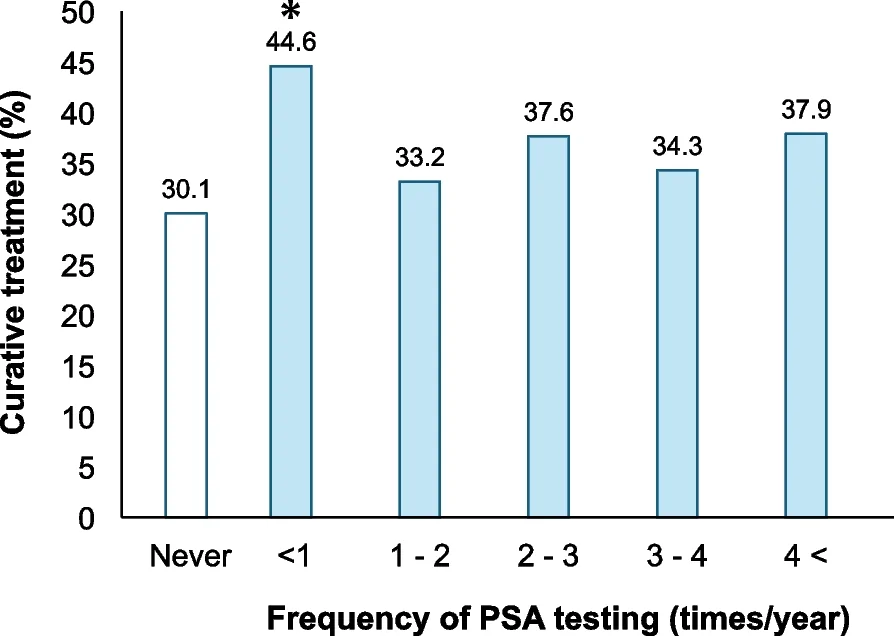
Proportion of Patients Receiving Curative Treatment According to the Frequency of PSA Testing. The proportion of patients who received curative treatment among each category of the annual frequency of PSA testing is shown. Abbreviation: PSA, prostate-specific antigen. *P* < 0.05 by chi-square tests with Bonferroni correction

Table 2 compares the baseline characteristics of patients who underwent PSA testing and those who did not. The frequency of PSA testing in patients who underwent PSA testing was 2.7 ± 1.6 times/year. Patients in the regular testing group were slightly but significantly older (75 ± 6.5 years vs. 74.3 ± 6.7 years, *p* < 0.001) and more likely to participate in Specific Health Checkups (40% vs. 29.6%, *p* < 0.001). The proportion of the patients who received curative treatment was significantly higher in the PSA testing group than in the non-PSA testing group (36.4% vs.30.1%, *p* < 0.001). The proportion of patients receiving curative treatment according to the frequency of PSA testing is shown in Fig. 2. Patients without PSA testing had the lowest proportion of curative treatment (30.1%), whereas patients undergoing PSA testing up to once per year showed higher proportions (44.6%, *p* = 0.049). Across categories of more frequent testing (1–2, 2–3, 3–4, and ≥ 4 times per year), the proportion of curative therapy remained consistently higher (33.2%–37.9%) than in patients without PSA testing, whereas there was no statistically significant difference.

**Table 2:** Characteristics of the patients with and without PSA testing. This table compares the baseline characteristics of elderly male patients with prostate cancer who had a history of treatment and underwent PSA testing (*n* = 737) with those who did not (*n* = 1,463). PSA testing was defined as one or more PSA tests performed more than six months before the initiation of prostate cancer treatment. The variables included age at diagnosis, comorbidities (hypertension, diabetes, and dyslipidemia), history of participation in the Specific Health Checkup program, annual PSA testing frequency, and treatment type (curative vs. non-curative). Non-curative treatment was further categorized into systemic therapy only and adjuvant or recurrent treatment. *p*-values were calculated to assess the differences between the groups

Variables	PSA testing (n = 737)	No PSA testing (n = 1463)	*p* value
Age at diagnosis of prostate cancer, years	75 ± 6.5	74.3 ± 6.7	0.009
Hypertension, *n* (%)	576 (78.1)	1089 (74.4)	0.058
Diabetes, *n* (%)	353 (47.9)	721 (49.3)	0.557
Dyslipidemia, *n* (%)	434 (58.9)	777 (53.1)	0.010
History of Specific Health Checkup, *n* (%)	295 (40)	433 (29.6)	< 0.001
PSA testing frequencies, *n*/year	2.7 ± 1.6	0 ± 0	< 0.001
Curative treatment, *n* (%)	268 (36.4)	440 (30.1)	0.003
Non-curative treatment, *n* (%)	469 (63.5)	1,023 (69.9)	

Continuous data is shown as mean ± standard deviation

*Abbreviation*: *PSA* prostate-specific antigen

### Characteristics of the patients with or without curative treatment

As shown in Table 3, the patients who received curative treatment had a significantly higher frequency of PSA testing than those who received non-curative treatment (1.01 ± 1.6 vs. 0.86 ± 1.6 times/year, *p* = 0.039). In patients who received curative treatment, the proportion of those who underwent PSA testing was greater than that of the patients who received non-curative treatment (37.9% vs. 31.4%, *p* = 0.003). Patients who participated in Specific Health Checkups were more frequent in the curative treatment group than in the non-curative treatment group (42.4% vs.28.7%, *p* < 0.001). Dyslipidemia tended to be more prevalent in the curative treatment group than in the non-curative treatment group (*p* = 0.054).

**Table 3:** Characteristics of the patients with curative treatment. This table presents a comparison of the baseline characteristics between treated prostate cancer patients who received curative treatment (n = 708) and those who did not (n = 1,492). The variables included the age at diagnosis, comorbidities (hypertension, diabetes, and dyslipidemia), a history of participation in the Specific Health Checkup program, the status of PSA testing (defined as PSA testing conducted more than six months prior to treatment initiation), and the annual frequency of PSA testing. *p*-values indicate the significance of the differences between the two groups

Variables	Curative treatment (*n* = 708)	Non-curative treatment (*n* = 1492)	*p* value
Age at diagnosis of prostate cancer, years	70.6 ± 5.2	76.5 ± 6.4	< 0.001
Hypertension, *n* (%)	499 (70.5)	1166 (78.2)	< 0.001
Diabetes, *n* (%)	328 (46.3)	746 (50)	0.110
Dyslipidemia, *n* (%)	411 (58.1)	800 (53.6)	0.054
History of Specific Health Checkup, *n* (%)	300 (42.4)	428 (28.7)	< 0.001
PSA testing, *n* (%)	268 (37.9)	469 (31.4)	0.003
PSA testing frequencies, *n*/year	1.01 ± 1.6	0.86 ± 1.6	0.039

Continuous data is shown as mean ± standard deviation

*Abbreviation*: *PSA* prostate-specific antigen

### Factors associated with curative treatment

**Fig. 3:**
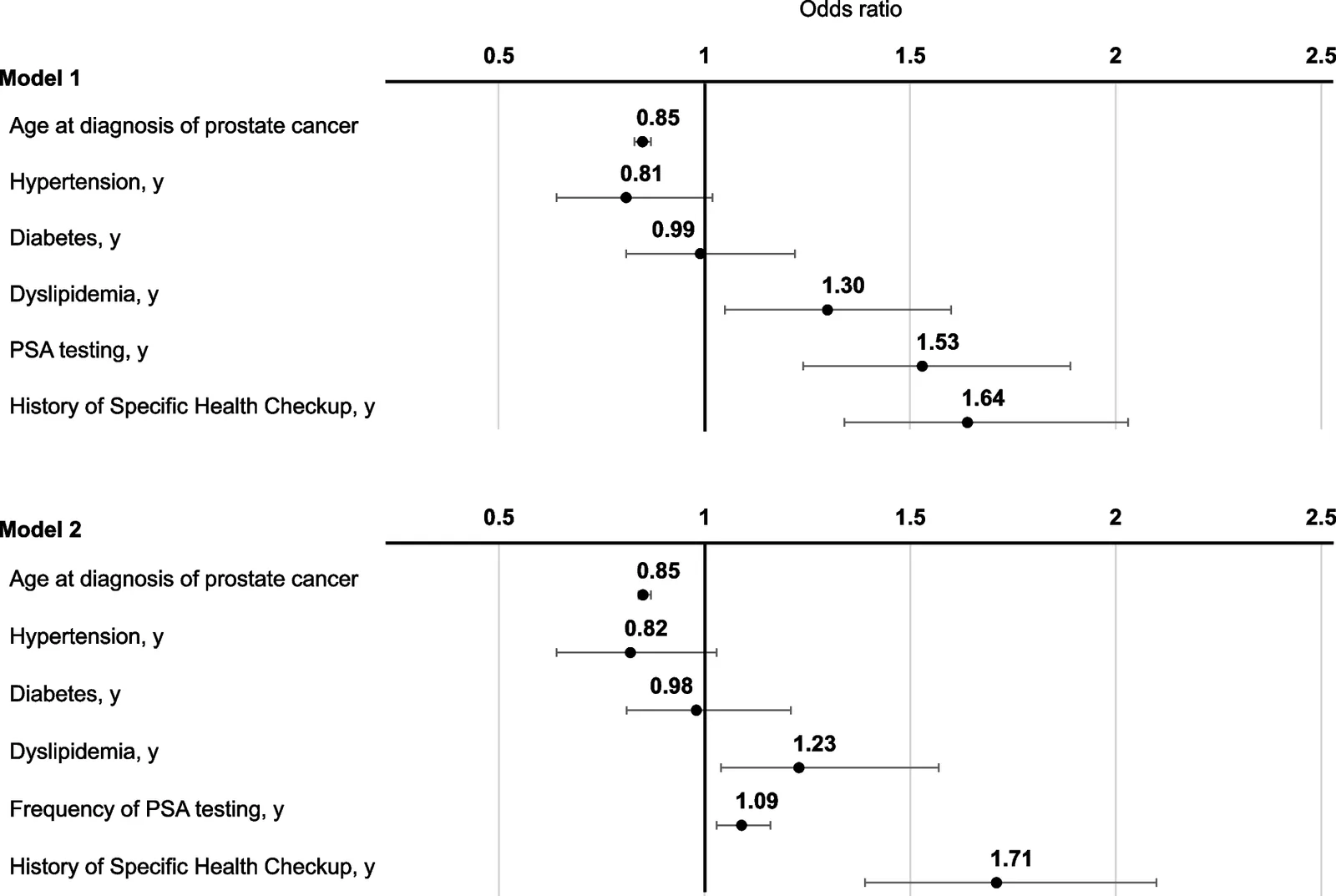
Factors associated with curative treatment. A multivariable logistic regression analysis was used to identify the factors associated with receiving curative treatment for prostate cancer. The variables included the age at diagnosis, the presence of hypertension, diabetes, dyslipidemia, history of participation in the Specific Health Checkup program, and PSA testing. Odds ratios (OR), 95% confidence intervals (C.I.), and *p*-values are shown for each factor. Abbreviations: PSA, prostate-specific antigen

In Fig. 3, a multivariable logistic regression analysis demonstrated that PSA testing (odds ratio (OR) = 1.53, 95% confidence interval (CI) = 1.24–1.89, *p* < 0.001) and PSA testing frequency (OR per additional test/year = 1.09, 95% CI = 1.03–1.16, *p* = 0.006) were independently associated with receiving curative treatment after adjusting for age at diagnosis, comorbidities, and participation in Specific Health Checkups (model 1). Participation in Specific Health Checkups was also a significant factor associated with curative treatment (Model 1: OR = 1.64, 95% CI = 1.34–2.03, *p* < 0.001; Model 2: OR = 1.71, 95% CI = 1.39–2.10, *p* < 0.001). An older age at diagnosis was inversely associated with the receipt of curative treatment, and dyslipidemia was positively associated with curative treatment. In Model 2, PSA testing frequency, analyzed as a continuous variable, was also independently associated with the receipt of curative treatment, thus supporting a dose–response–like relationship between surveillance intensity and treatment selection.

## Discussion

This population-based study using claims data from elderly men in Saga Prefecture demonstrated that PSA testing and higher PSA testing frequency were associated with an increased likelihood of receiving curative treatment for prostate cancer. These findings suggest that individuals who undergo PSA testing may be more likely to receive treatment with curative intent. However, this association should be interpreted cautiously and does not imply a causal relationship.

One of the most important considerations in interpreting our findings is the potential influence of selection bias and unmeasured confounding. Individuals who undergo PSA testing are likely to have higher health awareness, better access to healthcare, and more frequent interactions with medical providers. These factors may independently increase the likelihood of early diagnosis and subsequent curative treatment, regardless of the direct effect of PSA testing itself. This interpretation is supported by our finding that participation in Specific Health Checkups—screening programs that do not include PSA testing—was also independently associated with curative treatment.

Together, these findings suggest that healthcare engagement and health-seeking behavior may play a central role in determining treatment patterns.

Previous studies have reported that PSA testing practices are strongly influenced by healthcare systems, physician recommendations, and individual health awareness [10–12]. In addition, some studies have suggested that metabolic factors such as dyslipidemia or statin use may be associated with prostate cancer prognosis [13, 14].

Another critical limitation is the lack of detailed clinical information, such as tumor stage, Gleason score, and PSA levels at diagnosis. These factors are essential determinants of treatment selection and prognosis in prostate cancer. Without adjustment for disease severity, it is difficult to determine whether the observed association reflects earlier detection of less advanced disease among individuals undergoing PSA testing or simply differences in baseline disease characteristics. Therefore, the present findings should be interpreted as an association between PSA testing behavior and treatment patterns rather than evidence that PSA testing directly increases the likelihood of curative treatment.

The definition of PSA testing in this study was based on the presence of at least one PSA test more than six months prior to treatment initiation. Although this definition was intended to exclude diagnostic testing immediately preceding treatment, it may not fully capture the intensity or continuity of longitudinal PSA surveillance. To address this limitation, we additionally analyzed PSA testing frequency as a continuous variable and observed a consistent association with curative treatment, suggesting a possible dose–response–like relationship. However, this finding should also be interpreted with caution, given the observational nature of the study. Patients with longer observable periods before treatment may have had greater opportunities to undergo PSA testing, introducing the possibility of immortal time bias.

This study has several additional limitations. First, PSA testing performed outside the insurance system, such as self-paid screening, was not captured in the claims database, which may have led to misclassification of exposure. Second, residual confounding due to unmeasured factors such as socioeconomic status, educational level, and physician preference cannot be excluded. Third, multicollinearity among covariates was not formally assessed, although the variables included in the multivariable models were selected based on their clinical relevance. Fourth, the study population was limited to a single prefecture, which may affect the generalizability of the findings to other regions or healthcare settings.

Despite these limitations, this study provides valuable real-world evidence from a large population-based dataset in Japan. Our findings highlight that PSA testing behavior is closely associated with patterns of care and may reflect broader engagement with healthcare systems. From a public health perspective, these results suggest that improving access to healthcare services and promoting health awareness may be as important as the implementation of screening itself.

In conclusion, PSA testing was associated with a higher likelihood of receiving curative treatment for prostate cancer in this population. However, this relationship is likely influenced by underlying differences in healthcare engagement and patient characteristics rather than a direct causal effect of PSA testing. Further studies incorporating detailed clinical data and prospective designs are needed to clarify the impact of PSA testing on treatment decision-making and clinical outcomes.

## Supplementary Information

Supplementary Material 1.

## Data Availability

The datasets used and/or analyzed during the current study are not publicly available due to data use agreements but are available from the corresponding author on reasonable request.
