# Supplementary material for: PSA testing and receipt of curative treatment in elderly Japanese men with prostate cancer: a population-based study
Source: BMC Urol. 2026 Jul 27;26:254. doi: 10.1186/s12894-026-02266-3 (PMC13688820; doi:10.1186/s12894-026-02266-3)
Supplement: Supplementary file 1 [file 12894_2026_2266_MOESM1_ESM.docx]

Supplemental Table 1. Disease codes and name, billing code and treatment name, and medical intervention code and surgical procedure name for prostate cancer

| Disease code | Disease name | ICD10 |
| --- | --- | --- |
| 1859003 | Prostate cancer | C61 |
| 8848066 | Advanced prostate cancer | C61 |
| 8848043 | Localized prostate cancer | C61 |
| 8848040 | Castration-resistant prostate cancer | C61 |
| 8851347 | Metastatic castration-resistant prostate cancer | C61 |
| Medical intervention code | Surgical procedure name | K code |
| 150209310 | Surgery for malignant prostate tumor | K843-00 |
| 150326510 | Laparoscopic surgery for malignant prostate tumor | K843-02 |
| 150338810 | Laparoscopic-assisted small-incision surgery for malignant prostate tumor | K843-03 |
| 150390310 | Laparoscopic surgery for malignant prostate tumor (using endoscopic surgical support devices) | K843-04 |
| 180055010 | Carbon ion radiotherapy | M001-04 |
| 180055210 | Particle beam therapy | M001-04 |
| Billing code | Treatment name | Category Number |
| 620009249 | Ethinylestradiol | 247 |
| 640443027  640462004  642490105 | Goserelin | 249 |
| 610463172  621484703  621484705  622742500 | Flutamide | 429 |
| 620003534  622265601  620009411  620009412  620009413  620009416  620009417  620009420  620009421  620009422  620009423  621897501  621897504  621912301  621927301  621938701  621979301  622087503  622482001  622487201  622498101  622501501  622502701  622507101  622513701  622671401  622671501  622847300 | Bicalutamide | 429 |
| 620555101  620555201  620555301  620555401  621495301  622444901  622266501  622266601  622298301  622298401 | Leuprorelin | 249 |
| 620004939 | Estramustine | 421 |
| 622517201  622517301  622669301 | Degarelix | 249 |
| 622363801  622942001 | Abiraterone | 429 |
| 622606901  622607001 | Olaparib | 429 |
| 622623001  622623101 | Enzalutamide | 429 |
| 622489201 | Radium-223 chlorid | 429 |
| 622364601 | Cabazitaxe | 424 |
| 622676901 | Apalutamide | 429 |
| 622796301 | Darolutamide | 429 |
| Medical intervention code | PSA test | Category Number |
| 160037510 | PSA test | D009-00 |
| Medical intervention code | Procedure for prostate biopsy | Category Number |
| 190178410  190178510 | Prostate needle biopsy | A400-00 |
| 160098010 | Percutaneous needle biopsy | D412-00 |
| 160231810 | Prostate needle biopsy (MRI/ultrasound fusion imaging) | D413-00 |
| 160098110 | Prostate needle biopsy (other methods) | D413-00 |
